# Some Notes on the Nails

**Published:** 1890-11

**Authors:** John V. Shoemaker

**Affiliations:** Philadelphia, Pa.


### Some Notes on the Nails.

BY JOHN V. SHOEMAKER, A.M., M.D., PHILADELPHIA, PA.

Read in the Section of Dermatology and Syphiligrophy, at the forty-first annual meeting of the American Medical Association, at Nashville, Tenn., May, 1890.

In the early days of the world, before man had learn to utilize the stores of nature, the nails were of far more importance than at present. As the first weapon of attack and defense, the first implement of digging in the soil, or cutting or preparing the food, a healthy finger nail was one of the necessities for a healthy existence.

Even at the present time they are of the utmost importance as an ornamental and useful appendage of the skin.

They preserve the delicate and sensitive nerves of the posterior terminal portion of the fingers from contact with irritative substances. They protect the sense of touch in the tips of the fingers from becoming dull by constant contact, and perform a thousand other useful offices. It will thus be seen that attention to their health is of paramount importance. Fortunately for both physician and patient, diseases of the nails are comparatively limited in number. This is due to the fact that they are composed entirely of flattened epithelial scales which contain neither blood-vessels or nerves. They are, therefore, practically precluded from being the subject of either inflammation or neuralgia. They are, of course, liable to all forms of external injuries, but unless the injury extends to the structures beneath or adjacent to them, the result is not serious. The treatment of wounds or injuries to the nails is in fact a treatment of the structures which are wounded or injured at the same time. If the nail alone is fissured, or broken, or torn, there can be nothing done except to trim the surface down and wait for all trace of the injury to grow out, as it will do in all cases except when the matrix or root of the nail is destroyed.

In that event the nail will not be reproduced, but the integument will become hard and cicatricial like.

One of the diseases with which the nails are liable to be affected, and to which I wish to refer, is known as onychogryphosis, or hypertrophy of the nails.

This disease consists of an abnormal increase of either, or both, the length or thickness of the nail. It is due to an excessive proliferation of the cells of the root. It may be uniform, affecting the whole nail, or only certain portions. In the form most usually observed the hypertrophy or excessive cell deposits occur principally in the central portion of the toe nail, elevating it there and producing an upward curvature. As a result the edges of the nail are depressed, and forced into the soft structures of the toe, producing more or less irritation and inflammation. In neglected cases the inflammation may lead to very serious consequences, requiring, at times excision of the entire nail. This form of the disease is generally the result of prolonged pressure, as from tight fitting shoes, and is seldom observed anywhere except in the nails of the toes, especially the big toes. It is sometimes observed on the fingers in persons who from their occupation, or in obedience to fashion, are in the habit of wearing tight-fitting gloves. Idiopathic cases sometimes occur as the sequelæ of rheumatism, scarlet fever, and other diseases. This is, however, rarely noticed. The treatment of hypertrophy of the nail consists in removing the cause of the disease, and restoring the natural condition of the parts.

No remedies will do any permanent good if the cause be allowed to continue. If, as is usually the case, the disease is limited principally to the nail of the big toe and the adjacent soft parts, the patient must be ordered to remove the pressure, by either cutting out a piece from his ordinary foot covering, or to discard it for a slipper, until cured. When this is done the edges of the nail will no longer be forced down into the sensitive inflamed flesh, and relief will be given at once. The free edges of the nails should then be trimmed down as close as possible, and a mild sedative and astringent ointment applied to the painful parts. One of the best applications is a 5 per cent. ointment of the oleate of tin, or if there be much inflammation a 10 per cent. ointment of the oleate of lead.

Another excellent application is tannic acid 1 part, bismuth sub-nitrate 1 part, adipis 12 parts. If there be much pain 5 grains of powdered opium should be added to each ounce. In some cases the best results are obtained from applying pure carbolic acid directly to the raw surface. A momentary burning sensation is produced, which is quickly succeeded by entire relief from pain. A solid stick of nitrate of silver may be used to accomplish the same purpose. Another analgesic ointment is composed of salicylic acid 20 grains, ext. belladonna 10 grains, adipis 1 ounce. In chronic cases where the surface remains raw for days after the pressure has been removed, reparative action can usually be promptly produced by abandoning the use of ointments and dusting the surface with powdered cinchona bark, or pure tannic acid, or oxide of zinc, or bismuth sub-nitrate. If the nail remains thick, or continues to increase in thickness after the removal of all sources of pressure or irritation, its surface should be softened by frequent applications of liquor potassæ, and then gently scraped with a knife or abraded by a file until it returns to its natural condition. Nightly applications of salicylic acid ointment or plaster over the nails are also serviceable.

Internal medication is seldom required unless the pain be extremely severe, or there is some constitutional disease present. In the former instance full doses of morphia are indicated to allay pain and procure sleep. In the latter case appropriate anti-syphilitic, anti-strumous, anti-rheumatic, or other needed remedies should be freely given. If the hypertrophy is due to parasitic infiltration it may be necessary to destroy or remove the greater portion of the diseased nail surface.

---

Medical Achievement in China.—It is said of Dr. Kerr, a medical missionary at Canton, that he has in the past thirty-six years treated over 520,000 patients, and has prepared twenty-seven medical and surgical books. He has trained one hundred medical assistants, chiefly Chinese. China now possesses one hundred and four hospitals and dispensaries, at which, in 1889, more than 348,000 patients received treatment.

